# Thermoelectric, Electrochemical, & Dielectric Properties of Four ZnO Nanostructures

**DOI:** 10.3390/ma15248816

**Published:** 2022-12-09

**Authors:** Rusiri Rathnasekara, Grant Mayberry, Parameswar Hari

**Affiliations:** 1 Department of Physics and Engineering Physics, University of Tulsa, Tulsa, OK 74104, USA; 2 Oklahoma Photovoltaic Research Institute, University of Tulsa, Tulsa, OK 74104, USA

**Keywords:** thermoelectric, electrochemical, cyclic voltammetry, Seebeck coefficient, thermal conductivity, dielectric constant, impedance spectroscopy

## Abstract

In this work, we investigated the thermoelectric, electrochemical, and dielectric properties of four different ZnO morphologies, namely nanoribbons, nanorods, nanoparticles, and nanoshuttles. Temperature-dependent Seebeck coefficients were observed using thermoelectric measurements, which confirmed that all synthesized ZnO nanostructures are n-type semiconductors. The Van der Pauw method was applied to measure electrical conductivity, which was also used to calculate the thermal activation energy. Electrochemical properties were analyzed by cyclic voltammetry techniques under five different optical filters. Electrical conductivity of ZnO morphologies showed an increasing trend with increasing temperature. The highest electrical conductivity (1097.60 Ω^−1^ m^−1^) and electronic thermal conductivity ($1.16\times 10^{-4}$ W/mK) were obtained for ZnO nanorods at 425 K, whereas ZnO nanoshuttles carried the lowest electrical conductivity (1.10 $\times \;10^{-4}$ Ω^−1^ m^−1^) and electronic thermal conductivity (8.72 × 10^−7^ W/mK) at 325 K. ZnO nanorods obtained the maximum Power factor value in all temperature ranges. All nanostructures showed electro-catalytic performance with different optical filters. From impedance spectroscopy analysis, ZnO nanorods showed the highest dielectric constant at high frequencies (>1 MHz) at 2.02 ± 0.06, while ZnO nanoshuttles gave the highest dielectric constant at low frequencies (<100 Hz) at 9.69 ± 0.05. These results indicate that ZnO nanorods have the most favorable thermoelectric, electrochemical, and dielectric properties compared to all other ZnO morphologies.

## 1. Introduction

Among various semiconductor nanomaterials, Zinc Oxide (ZnO) has been a leading candidate for device applications especially due to its eco-friendly nature, chemical stability, advanced optical properties, non-toxicity, cost-effectiveness, etc. [1,2,3]. Wurtzite ZnO is an n-type semiconductor with a wide bandgap of 3.37 eV and a large free exciton binding energy of about 60 meV [4]. ZnO nanomaterials have a wide range of applications in medical and industrial (biosensors, gas sensors, solar cells, LED, filters, lasers, etc.) [5,6,7,8,9,10].

N-type ZnO nanomaterials have the potential for thermoelectric applications due to their high Seebeck coefficient value (~−400 μVK^−1^), low electrical resistivity, high melting points, non-toxic nature, and stability behavior at high temperatures [11,12,13,14,15,16,17,18]. Thermoelectric materials are investigated for environmentally friendly applications such as conversion of waste heat into electrical energy [19,20,21]. The performance of thermoelectric properties of a material is mainly dependent on the materials’ Seebeck coefficient, electronic conductivity, electronic thermal conductivity, lattice thermal conductivity, and temperature [22,23,24]. Hence, it is important to investigate the thermoelectric parameters of different ZnO morphologies as outlined in this work.

Conventionally, ZnO nanoparticles have been used as electrochemical biosensors to identify active ingredients in biological specimens [25]. Electroactive device responses are analyzed by electrochemical techniques [26,27]. Cyclic Voltammetry is the most prominent electrochemical technique for investigating kinetics and electron transfer in nanomaterials. It is a widely used characterization technique for studying electrochemical behavior of electrodes. Cyclic Voltammetry is also one of the most accurate techniques to determine the bandgap of materials and is widely used to characterize dye-sensitized solar cells [28,29]. In this study, we use Cyclic Voltammetry to elucidate the difference between various ZnO morphologies.

It is important to know the real and complex dielectric constant of a semiconductor material when designing devices. The dielectric constant governs AC response as well as the width of the depletion region in a semiconductor junction. Optically, the dielectric constant is directly related to the complex index of refraction of a nonmagnetic material and the skin depth of a conductive material. For photovoltaic applications, the dielectric constant governs the rate of recombination losses in the absorbing layer, and a higher real dielectric constant leads to a slower recombination rate and therefore higher efficiency [30]. The same is true for thermoelectric materials; a thermoelectric material with a higher dielectric constant will be more efficient due to lower recombination losses. Previous literature shows that in extreme cases the morphology of a nanomaterial can cause the dielectric constant to vary by multiple orders of magnitude [31]. Thus, a measurement of the dielectric constant of various ZnO morphologies is crucial to assess their usefulness in photovoltaic and thermoelectric applications. In addition, dielectric spectra give insight into the material properties. A material with low surface conductivity will exhibit high ionic-interfacial polarization, and this is often reflected in the dielectric spectra as a large increase of the real permittivity at lower frequencies [32]. It is not uncommon for the dielectric spectra of a resistive nanomaterial to reflect that of a series of two lossy capacitors and show three polarization regimes at low frequencies, due to the buildup of charges on the many small surfaces. In contrast, the dielectric constant in the MHz range is more indicative of the dielectric behavior inside the material itself rather than on the surface, and it is more relevant when considering recombination losses.

ZnO nanomaterials can be grown through several techniques, such as chemical bath deposition (CBD), chemical vapor deposition (CVD), sole-gel, microwave-assisted techniques, precipitation methods, etc. [33,34,35,36]. Depending on synthesized conditions, chemicals, and methods, ZnO has been fabricated with various morphologies, namely, nano springs, nanoribbons, nanorods, nanoparticles, nanoshuttles, nanotubes, nanorings, nanoflowers, nanobelts, etc. [33,37,38,39]. Morphology and nanostructure size are some of the key parameters controlling the physical, thermal, and chemical properties of nanomaterials. For instance, in 2022, Doustkhah et al. have synthesized ZnO morphologies (rod-like structure) by controlling treatment temperature and choosing appropriate structure-directing agent [40]. In our previous work, we have demonstrated the optical, electrical, and wetting properties of four different ZnO morphologies, namely nanoribbons, nanorods, nanoparticles, and nanoshuttles [33].

In this work, we report the results of thermoelectric, electrothermal, and dielectric measurements on four different morphologies, namely, nanoribbons, nanorods, nanoparticles, and nanoshuttles. These morphologies were synthesized by using chemical bath deposition and microwave methods. Thermoelectrical properties of morphologies were analyzed to yield the Seebeck coefficient, electrical conductivity, electronic thermal conductivity, total thermal conductivity, power factor, figure of merit values, and activation energy from 325 K to 425 K. Cyclic voltammetry characterization was employed to study the electrochemical properties of different morphologies under five different optical filters (red, orange, yellow, green, and blue). The dielectric properties of different morphologies were determined by impedance spectroscopy from 100 Hz to 5.1 MHz. Our results are described in Section 3.

## 2. Materials and Methods

### 2.1. Materials

Zinc acetate dehydrates (Zn (CH_3_COO)_2_·2H_2_O, >99.50%), Zinc nitrate hexahydrate (Zn (NO_3_)_2_ 6H_2_O, >99.5%), Hexamethylenetetramine (C_6_H_12_N_4_, >98%), Sodium hydroxide (NaOH, >97%), Ammonium hydroxide (NH_4_OH, 28–30%), Ethyl alcohol (C_2_H_6_O, >98%), Potassium iodide (KI >99.00%), Iodine (I_2_ >99.99%), Acetic acid (CH_3_CO_2_H, ≥99%), and Triton X-100 (t-Oct-C_6_H_4_-(OCH_2_CH_2_)_x_OH, x = 9–10) were purchased from Sigma Aldrich (St. Louis, MO, USA).

### 2.2. Preparation of ZnO Nanostructures and Photoanode

Detailed preparation methods of ZnO nanostructures, namely, nanoribbons, nanorods, nanoparticles, nanoshuttles, and photoanodes are found in our previous work [33]. In this work, all four ZnO nanostructures were post-annealed at 300 °C for one hour.

### 2.3. Preparation of Electrolyte

The electrolyte solution was obtained by dissolving 0.05 M Iodine ($I_{2})$ and 0.5 M of Potassium iodide (KI) in 100 mL of acetonitrile and sonicated for 3 h [41].

### 2.4. Material Characterization

The electrochemical behavior of four different nanostructured ZnO morphologies (nanoribbons, nanorods, nanoparticles, and nanoshuttles) with different optical filters (red, orange, yellow, green, and blue) was characterized by using Cyclic Voltammetry (C-V) techniques (Ossila, Sheffield, UK, T2006A). The C-V measurements were taken 5 different times for each morphology. In the C-V system, Platinum wire, Ag/AgCl electrode, and prepared different photoanodes were assigned as a counter electrode, reference electrode, and working electrode, respectively.

All electrothermal measurements were analyzed within the 325 K to 425 K temperature range and measurements were repeated 10 different times for each morphology. Seebeck coefficients were analyzed through the Seebeck controller technique (MMR Technologies, San Jose, CA, USA). The Van der Pauw method was employed (MMR Technologies) to obtain electrical conductivity data. Based on both the Seebeck coefficient values and the electrical conductivity values, the electronic thermal conductivity, power factor, figure of merit, and activation energy were derived for all morphologies.

The dielectric properties of four different nanostructures were analyzed by impedance spectroscopy from 100 Hz to 5.10 MHz (Zurich Instruments, Zurich, Switzerland, MFIA). For the nanoribbon, nanorod, and nanoshuttle samples, the dielectric constants were measured directly on the slides. Two slides of the desired material were placed 20 mm apart with an FTO glass slide placed on top face-down bridging the two. A 0.50 kg weight was placed on top of the FTO glass slide to promote contact between the material and the slide. Impedance spectra were taken between 100 Hz and 5.10 MHz. Separation distances ranged from 20 mm to 6 mm with 2 mm steps. The area of contact was determined using ImageJ software and the thickness of the material was determined from SEM images, where the morphologies were also verified. The thicknesses were measured for each morphology at 20 different locations on the slides using SEM and ImageJ. There were two slides of each material, measured at 5 different separation distances within the 20 mm to 6 mm range, effectively meaning 10 measurements. The numerous amounts of nanostructures on each slide add reproducibility. For the nanoparticle samples, small volumes of powder were inserted sequentially into a dielectric cell and impedance spectra in the 100 Hz–5.1 MHz range were taken. This dielectric cell was normalized to the dielectric constant of water, similar to [42], so geometry was unimportant. Five volumes of particles were tested, and this data showed especially high agreement.

## 3. Results

### 3.1. Cyclic Voltammetry Analysis

**Figure 1:**
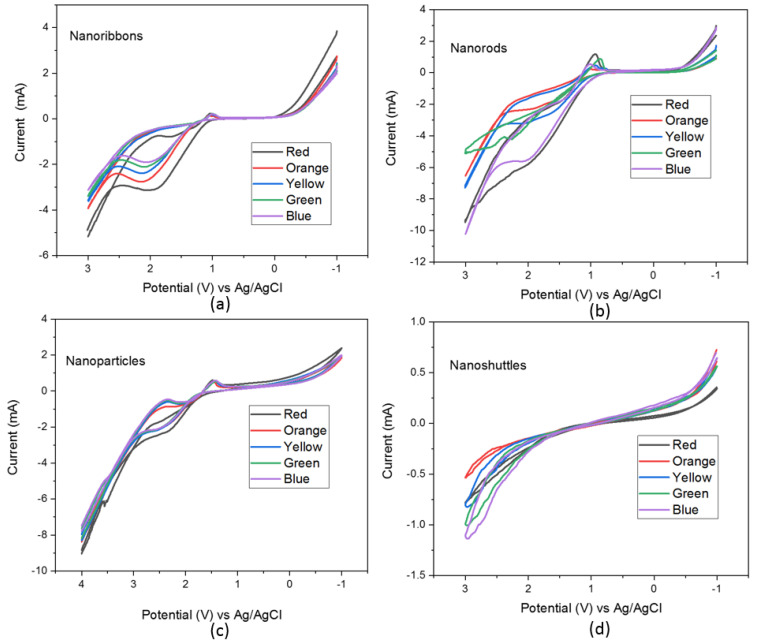
C-V spectra of ZnO nanostructures (**a**) nanoribbons, (**b**) nanorods, (**c**) nanoparticles, and (**d**) nanoshuttles.

Electrochemical properties of different ZnO nanostructure electrodes were studied through Cyclic Voltammetry (C-V) characterization techniques under five different optical filters. Table 1 lists the wavelength variation of five different optical filters which are red, orange, yellow, green, and blue. All five filters were employed in C-V measurements to obtain monochromatic wavelengths. The C-V graphs of all ZnO nanostructures, namely, nanoribbons, nanorods, nanoparticles, and nanoshuttles, are shown in Figure 1. All C-V measurements were taken for four different ZnO morphologies’ working electrodes in a solution containing $I^{-}/I_{3}\;$electrolyte at a scanning rate of 100 mV/s.

The C-V graphs’ oxidation and reduction peak positions were employed to calculate the highest occupied molecular orbital (HOMO), the lowest unoccupied molecular orbital (LUMO), and bandgap energy levels. Based on the HOMO (Equation (1)), LUMO (Equation (2)), and bandgap (Equation (3)) energy levels, we have studied the variation of electrochemical properties concerning morphologies and different monochromatic wavelengths [43,44].

(1) $$E_{HOMO\;}=-e\left[E_{oxidation}^{onset}+4.4\;\right]$$

(2) $$E_{LUMO}=-e\left[E_{reduction}^{onset}+4.4\;\right]$$

(3) $$E_{g\;}=E_{LUMO}-E_{HOMO}$$

where, $E_{HOMO}$ is the energy level of HOMO, $E_{LUMO}$ is the energy level of LUMO, $E_{oxidation}^{onset}$ is the oxidation onset value, $E_{reduction}^{onset}$ is the reduction onset value, −4.4 eV is the value of the adjustment factor, and $E_{g}$ is the bandgap energy.

**Figure 2:**
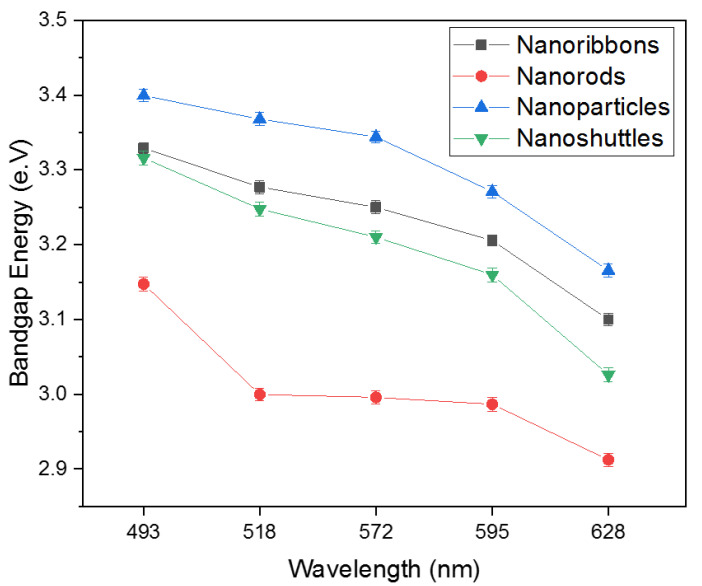
Variation of bandgap energy with different wavelengths.

All ZnO morphologies show electrochemical performance corresponding to the different optical filters. All C-V graphs’ oxidation and reduction peaks demonstrated an anodic and cathodic behavior in different morphologies. The key parameters of the C-V analysis were recorded in Table 2. The C-V results suggest that the position of the HOMO and LUMO energy levels are considerably varied concerning morphology and the choice of optical wavelength used for excitation. Additionally, we noted that for all morphologies, the longer wavelength improved the redox reaction. As a result, the calculated bandgap of all samples decreased with an increasing wavelength of optical excitation (Figure 2). This decreasing trend may be attributed to the absorption from defect levels of ZnO such as Oxygen-related defects, and Zn defects. All ZnO nanostructures have shown the lowest bandgap value with a red optical filter and the highest bandgap value with a blue optical filter. These results suggested that the optical properties of the ZnO nanostructure is dependent on its morphology.

### 3.2. Thermoelectric Analysis

**Figure 3:**
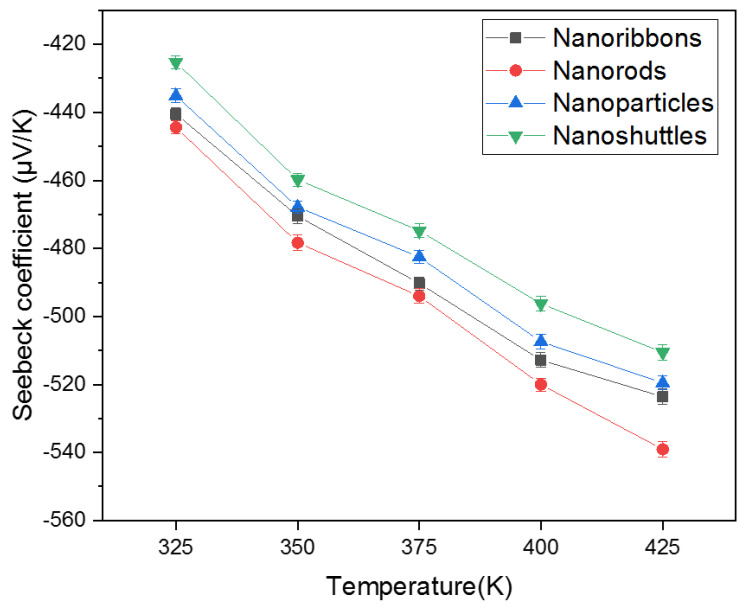
Temperature-dependent Seebeck coefficient of ZnO nanoribbons, nanorods, nanoparticles, and nanoshuttles.

**Figure 4:**
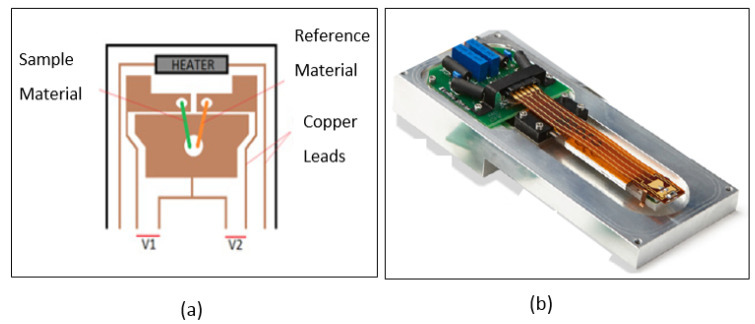
Commercial system (Seebeck thermal stage) of MMR Technologies Inc. (Figures are redrawn from [45] (**a**) Seebeck stage which has two pairs of thermos couples: green line is sample material and orange line is reference material. (**b**) MMR refrigerator which controls the temperature.

Figure 3 shows the temperature-dependent Seebeck coefficient measurements of four different morphologies (nanoribbons, nanorods, nanoparticles, and nanoshuttles). Seebeck coefficient values were obtained from the temperature of 325 K to 425 K by using Seebeck controller techniques. Figure 4 describes the commercial system (Seebeck thermal stage) of MMR Technologies Inc. [45]. It provides the temperature dependence of the Seebeck voltage for different materials (metals and semiconductors). We used the width of sample as 1 mm, and length as 5 mm. Results revealed that all morphologies’ Seebeck coefficients were negative. This negative sign confirmed that all synthesized ZnO nanostructures were n-type semiconductors. When we consider the absolute value of the Seebeck coefficient, which increased with increasing temperature for all morphologies, the highest absolute value of the Seebeck coefficient was obtained by nanorods while the lowest value was shown by nanoshuttles for all temperature values. Correspondingly, the magnitude of the Seebeck coefficient of ZnO nanorods increased from 444.44 µVK^−1^ to 539.08 µVK^−1^, taking these values from 325 K to 425 K. The nanoshuttles’ Seebeck coefficient changed in magnitude from 425.29 µVK^−1^ to 510.59 µVK^−1^ when the temperature increased from 325 K to 425 K. However, we have observed that Seebeck coefficients did not significantly change from one morphology to another. 

**Figure 5:**
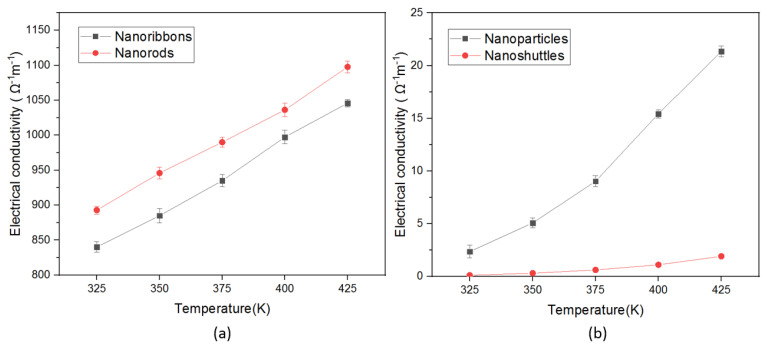
Temperature-dependent electrical conductivity of ZnO nanostructures (**a**) nanoribbons, and nanorods, (**b**) nanoparticles, and nanoshuttles.

The electrical conductivity of four different ZnO nanostructures were measured by the Van der Pauw techniques. Figure 5 indicates the electrical conductivity variation of all nanostructures from the 325 K to 425 K temperature range. In this analysis, we noted that electrical conductivity values considerably change with nanostructure morphology and surface temperature. When the temperature increased from 325 K to 425 K range, the electrical conductivity values also show an increasing pattern, which is a typical semiconducting behavior [21]. Both nanorods and nanoribbons have obtained significantly higher electrical conductivity values compared to nanoparticles and nanoshuttles. The nanorod morphology produced a higher conductivity value, which may be due to the higher aspect ratio of rod shape, and the higher crystallinity nature of rod morphology [33]. The lowest conductivity was obtained by ZnO nanoshuttles at all temperature ranges. This may be attributed to the strong agglomeration and low crystalline nature of the nanoshuttle morphology [11]. The surface temperature also enhanced the charge carrier flow rate, which caused the conductivity values to rise [46]. These results suggest that morphology and surface temperature are the main control parameters of electrical conductivity in nanostructured ZnO. It may be attributed to the different grain boundary properties, grain sizes, and aspect ratio effects corresponding to different morphologies [47,48].

**Figure 6:**
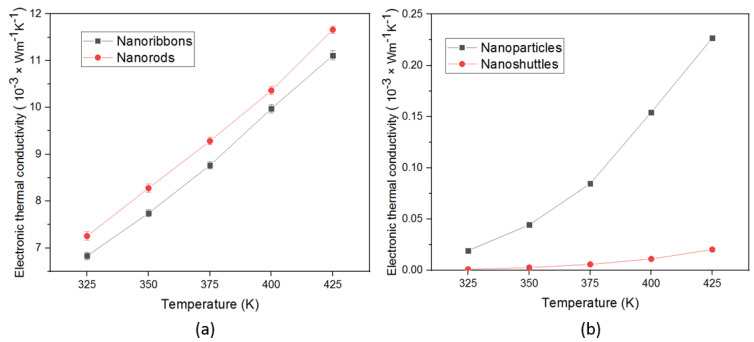
Temperature-dependent electronic thermal conductivity of ZnO nanostructures (**a**) nanoribbons, and nanorods, (**b**) nanoparticles, and nanoshuttles.

The variation of electronic thermal conductivity ($K_{e}$) of four different ZnO nanostructures is shown in Figure 6. We have observed that electronic thermal conductivity in all morphologies enhanced with increasing the surface temperature from 325 K to 425 K. The main contribution to the enhancement of electronic conductivity values of morphologies come from the electronic part not the phonon component. Electronic thermal conductivity was determined by using the Wiedemann—Franz relation (Equation (4)).

(4) $$\;\;\;K_{e}=LT\sigma \;\;\;$$

where *L* represents the Lorentz number, T is temperature, and σ is the electrical conductivity [21]. Overall, we observed extremely low electronic thermal conductivity values for all morphologies. This may be due to the effect of the carrier scattering near grain boundaries and the structural defects of nanostructures.

**Figure 7:**
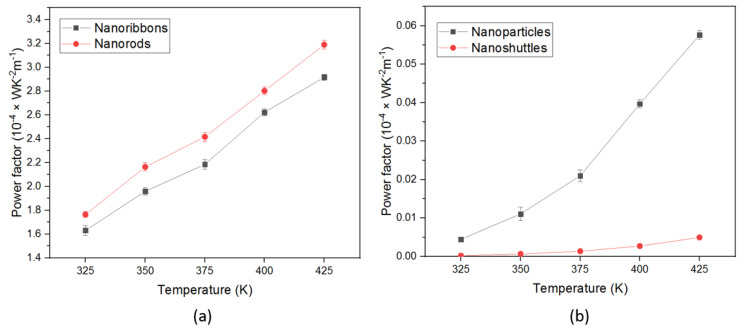
Temperature-dependent power factor of ZnO nanostructures (**a**) nanoribbons, and nanorods, (**b**) nanoparticles, and nanoshuttles.

The performance of thermoelectric materials can be also assessed using power factor (*PF*) values. The power factor, which combines the electrical conductivity and Seebeck coefficient, is indicated in Equation (5). Figure 7 shows the power factor variation in all morphologies with temperature. According to this analysis, the maximum power factor value of 3.19 $\times 10^{-4}$ WK^−2^ m^−1^ was achieved by nanorods compared to all nanostructures, followed by nanoribbons 2.87 $\times 10^{-4}$ WK^−2^ m^−1^ at 425 K. The lowest power factor value obtained in nanoshuttles of 1.94 $\times 10^{-8}$ WK^−2^ m^−1^ at 325 K. The enhancement of the power factor values is due to the higher electrical conductivity and higher Seebeck coefficient values of nanorods and nanoribbons. Therefore, we may conclude that nanorods and nanoribbons are the most appropriate nanostructures for thermoelectrical applications. In addition, we obtained an increasing trend of power factor with increasing temperature in all morphologies.

(5) $$PF=\sigma S^{2}\;$$

where σ is electrical conductivity, and S is the Seebeck coefficient.

**Figure 8:**
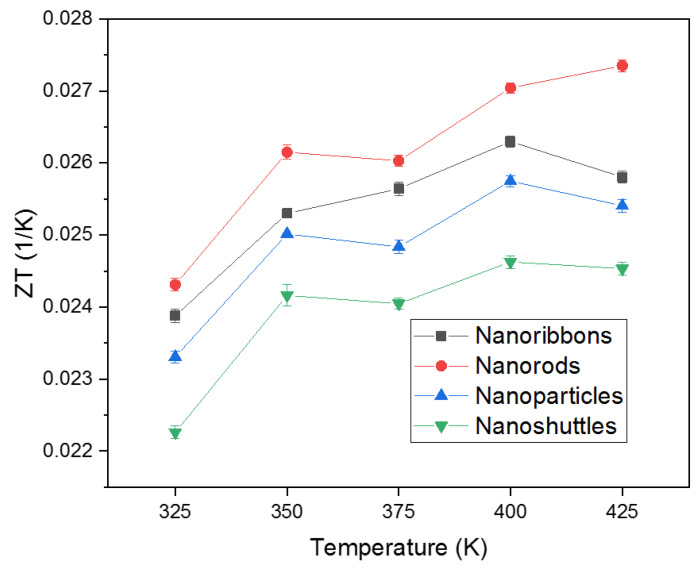
Figure of merit of ZnO nanostructures.

The thermoelectric figure of merit (ZT) of four different ZnO nanostructures are shown in Figure 8. The ZT values represent the efficiency of the thermoelectric material (Equation (6)).

(6) $$ZT=\frac{S^{2}\sigma }{K_{Total}}\;\;\;\;$$

Here, $S,\;\sigma ,\;K_{Total}\left(K_{Total}=K_{e}+K_{l}\right)$ are the Seebeck coefficient, electrical conductivity, and total thermal conductivity, respectively [22]. The heat transport in a material can be expressed in terms of electron movement and lattice vibrations. Therefore, the total thermal conductivity ($K_{Total\;})\;$is a sum of electronic thermal conductivity ($K_{e}$) and lattice thermal conductivity $(K_{l}$). According to the principle of the figure of merit, high efficiency can occur with high S and σ values and low $K_{Total}$ values. Previous studies show that thermal conductivity measurements in ZnO nanostructures exhibit low thermal conductivity compared to bulk samples [49,50,51,52,53]. This reduction in thermal conductivity in ZnO nanostructures is mainly due to the high surface-to-volume ratio of nanostructures compared to bulk samples [54]. Nanostructures contain higher atomic densities on the surface. Due to high surface densities, nanostructures show an enhancement in the surface scattering of phonons which results in a reduction of the phonon mean free path. As a result, in nanostructures, due to low mean free path, the lattice contribution of thermal conductivity is low compared to the electronic component. In addition, the boundary scattering of phonons in nanostructure domains is attributed to the effect of surface specular reflection [54,55,56]. Berman et al. have observed a decreasing trend in specular reflection with temperature, leading to a reduction in lattice (phonon) contribution to thermal conductivity [57]. According to possible phonon scattering mechanisms evaluated in nanostructures, the contribution of lattice thermal conductivity of nanostructured compounds is about 2% of the total thermal conductivity [58]. Therefore, in order to calculate the figure of merit (ZT), we are justified in ignoring the lattice contribution to the thermal conductivity [23,59]. Among all ZnO morphologies, the highest ZT value obtained by ZnO nanorods was 2.73 $\times \;10^{-2}$K^−1^ at 425 K, while the lowest value obtained by ZnO nanoshuttles was 2.22 $\times \;10^{-2}$K^−1^ at 325 K. Changes in this ZT values mainly depend on the variation of Seebeck coefficient, electrical conductivity, and thermal conductivity values.

**Figure 9:**
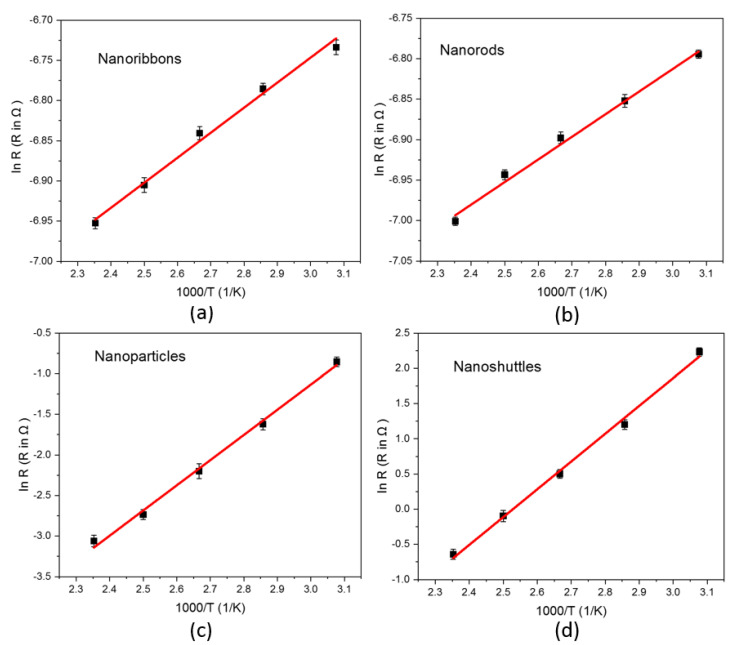
ln R versus 1000/T Arrhenius plot of ZnO nanostructures (**a**) nanoribbons, (**b**) nanorods, (**c**) nanoparticles, and (**d**) nanoshuttles. The red line indicates the linear Arrhenius plot.

Figure 9 represents the Arrhenius plot of four different morphologies. The Arrhenius plot represents the variation of the electrical resistance of semiconducting materials as a function of inverse temperature (Equation (7)). The electrical resistivity of semiconductors is also significantly varied corresponding to defects of nanostructure morphology such as impurities, vacancies, and other [60].

(7) $$R\left(T\right)=R_{0\;\;}e^{\frac{∆E}{k_{B}T}}\;\;$$

where R and $\;R_{0}$ are electrical resistivity at temperatures T and 0 K, respectively, ∆E represents the activation energy, $k_{B}$ is the Boltzmann constant, and *T* is the temperature [61]. Using the slope of the Arrhenius plot, we calculated the activation energy in each nanostructure. The Seebeck coefficient values confirmed that all synthesized ZnO nanostructures are n-type semiconductors. The activation energy extracted from electrical conductivity corresponds to the energy difference between the bottom edge of the conduction band and the donor level. We determined the activation energy of nanoribbons, nanorods, nanoparticles, and nanoshuttles as 2.68 $\times \;10^{-2}$ eV, 2.40 $\times \;10^{-2}$ eV, 2.67 $\times \;10^{-1}$ eV, and 3.40 $\times \;10^{-1}$ eV, respectively. The variation of activation energy may be attributed to changes in the potential barriers and defect (e.g., donor) states of four different ZnO nanostructures [60]. The lowest activation energy was obtained in ZnO nanorods, which have the highest conductivity values in all temperature ranges. From the previous study by Sakellis, we can attribute lower activation energies to lower activation volumes, which mainly correlated to physical defects [60]. Therefore, ZnO nanorods morphology has the fewer dominant defects compared to other ZnO morphologies.

**Figure 10:**
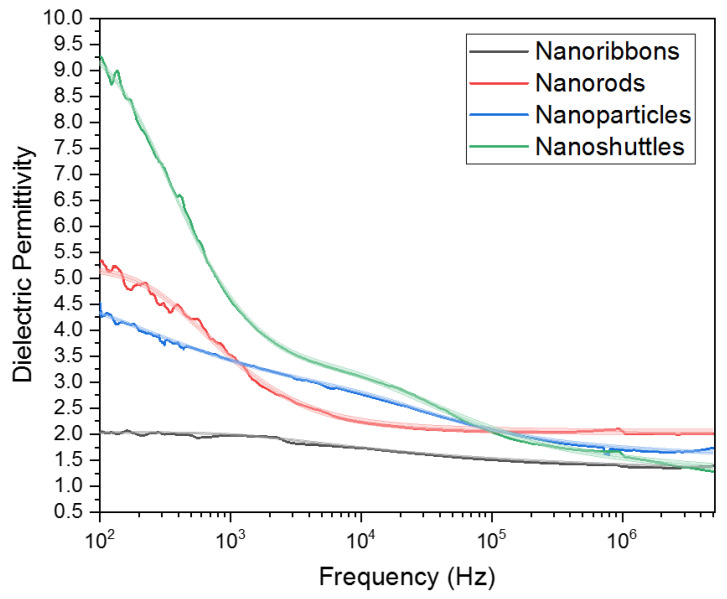
Dielectric spectra of ZnO nanostructures.

The dielectric spectra of each of the four ZnO morphologies are shown in Figure 10. The impedance spectra from 1 MHz–5.1 MHz were fitted to a transmission line model [42] in Z-fit, and the series inductive reactance and resistance values were subtracted. From this, the complex dielectric constant at each frequency can be calculated by using Equation (8).

(8) $$\;A\varepsilon^{*}=\frac{d}{j\omega }{\left(Z-R_{s}-j\omega L\right)}^{-1}\;\;\;$$

where d is the thickness or depth of the material; Z is the measured impedance; $R_{s}$ is the series resistance; L is the series inductance; $\varepsilon^{*}$ is the complex dielectric constant of the sample, the dependent variable; and A is the area of contact, the independent variable.

For nanoparticles, the series inductive reactance and resistance were also subtracted. The resulting equivalent circuit is a lossy capacitor and capacitance can be solved directly at each frequency. The real dielectric constant was determined at each frequency by comparing the volumetric capacitive dependence of the nanoparticles with that of water (Equation (9)) [42].

(9) $$\;\;\;\;\varepsilon^{\prime }=\frac{m}{m_{w}}\left(\varepsilon_{w}-\varepsilon_{a}\right)+\varepsilon_{a}\;\;\;\;\;$$

where m is the volumetric capacitive dependence at each frequency, $m_{0}$ is the volumetric capacitive dependence of water, $\varepsilon_{w}$ is the dielectric constant of water (80.42), and $\varepsilon_{a}$ is the dielectric constant of air (1.0006). The resulting real dielectric spectra were fit to the Cole-Davidson model described by Equation (10) [62].

(10) $$\;\;\varepsilon^{\prime }=Re\left(\varepsilon_{\infty }+\frac{\varepsilon_{s}-\varepsilon_{\infty }}{{\left(1+j\omega \tau \right)}^{\beta }}\right)=\varepsilon_{\infty }+\frac{\varepsilon_{s}-\varepsilon_{\infty }}{{\left(1+\omega^{2}\tau^{2}\right)}^{\frac{\beta }{2}}}\cos\left(\beta \tan^{-1}\omega \tau \right)$$

where$\;\varepsilon_{s}\;$is the low frequency dielectric response of the material,$\;\varepsilon_{\infty }$ is the high frequency dielectric response, τ is the relaxation time for the dielectric polarization mechanism in question (in this case ionic polarization), and β is a fitting parameter relating to the narrowness of the transition region of the dielectric constant with respect to frequency. The Cole-Davidson parameters are listed in Table 3 for nanoribbons and nanorods.

The dielectric spectra of nanoparticles and nanoshuttles both exhibited multiple curves, although it is more apparent in the latter. Additional terms can be added to the ColeDavidson model and so (Equation (11)) was used to reflect this behavior. Recall that this is expected behavior for materials where ionic polarization is due to the buildup of charge on many surfaces.

(11) $$\varepsilon^{\prime }=\varepsilon_{\infty }+\frac{\varepsilon_{i}-\varepsilon_{\infty }}{{\left(1+\omega^{2}{\tau_{i}}^{2}\right)}^{\frac{\beta_{i}}{2}}}\cos\left(\beta_{i}\tan^{-1}\omega \tau_{i}\right)+\frac{\varepsilon_{s}-\varepsilon_{i}}{{\left(1+\omega^{2}{\tau_{s}}^{2}\right)}^{\frac{\beta_{s}}{2}}}\cos\left(\beta_{s}\tan^{-1}\omega \tau_{s}\right)\;$$

$\beta_{s}$ and $\tau_{s}\;$serve the same function as *β* and *τ* in Equation (10), and $\beta_{i},\;\tau_{i}\;,\;\varepsilon_{i}$ define similar parameters for a second polarization curve. $\varepsilon_{i}\;$acts as an “intermediate” dielectric constant between the two polarization curves. The Cole—Davidson parameters are listed in Table 4 for nanoparticles and nanoshuttles.

The end behaviors of each morphology can be found in Table 3 and Table 4. *ε_s_* corresponds to the low frequency dielectric constant and *ε_∞_* corresponds to the high frequency dielectric constant. According to our calculations, ZnO nanorods have the highest dielectric constant at high frequencies (>1 MHz) at 2.02 ± 0.06, followed by nanoparticles at 1.42 ± 0.03, nanoribbons at 1.21 ± 0.02, and nanoshuttles at 1.15 ± 0.06. ZnO nano shuttles have the highest dielectric constant at low frequencies (<100 Hz) at 9.69 ± 0.11, followed by nanorods at 5.27 ± 0.06, nanoparticles at 4.68 ± 0.01, and nanoribbons at 2.01 ± 0.02.

## 4. Discussion

This paper presents results from thermoelectrical, electrochemical, and dielectric properties of four different ZnO morphologies which are nanoribbons, nanorods, nanoparticles, and nanoshuttles. Electrochemical properties of different ZnO morphologies electrodes were studied by Cyclic Voltammetry (CV) techniques under five different optical filters (Figure 1). All C-V graphs show that the electrocatalytic performance with different optical filters. The method of C-V study established the fact that redox reactions can be varied concerning the morphology of ZnO nanostructure and the choice of optical wavelength used for excitation. We used the C-V characterization method to determine the HOMO energy level, LUMO energy level, and bandgap of nanostructures. All nanostructure C-V graphs have demonstrated peaks that are related to the anodic and cathodic behaviors of the nanostructure-based electrode. We determined the HOMO and LUMO energy levels by comparing the oxidation and reduction peak positions. Based on these values, we also calculated the bandgap of the nanostructures (Figure 2). All ZnO morphologies recorded the lowest bandgap under a red filter, whereas the highest bandgap was observed with a blue filter. All samples’ HOMO and LUMO energy levels changed concerning the morphology and optical filter wavelength. Generally, the oxidation peak current increased with increasing the optical filter wavelength and the oxidation peak slightly shifted in the negative direction with increasing optical filter wavelength. Furthermore, we noted that, from the C-V spectra for all morphologies, longer wavelengths improved the redox reaction. As a result, the bandgap of all samples decreased with an increasing wavelength of optical excitation. This decreasing bandgap of ZnO may be attributed to the absorption from defect levels in ZnO such as Oxygen-related defects, and Zn defects. Different morphologies of ZnO indicated significantly different optical properties. For all optical filters, nanorods show the most remarkable current response compared with other nanostructures. The enhancement in current intensity demonstrates the superior electrochemical performance of the ZnO nanorods. It may be attributed to the increase in the electron transfer rate and conductivity of the nanorods’ morphology, and the good crystallinity nature of the morphology.

In this work, we also demonstrated the influence of temperature—dependent thermoelectric properties of four different ZnO morphologies. All results are presented with the temperature range from 325 K to 425 K. The thermoelectric properties of a material can be interpreted by the figure of merit, which involves the Seebeck coefficient, the electrical conductivity, and the total thermal conductivity. All ZnO morphologies show an enhancement in the thermoelectric properties with different values. We observed that the Seebeck coefficients of all morphologies were negative across the temperature range, which confirmed that all synthesized ZnO nanostructures are n-type semiconductors (Figure 3). Therefore, all ZnO morphologies have electrons as the leading contributor in the transport properties. The absolute value of Seebeck coefficients increased with increasing the temperature. This improvement in Seebeck coefficient values leads to an improvement in the thermoelectric properties of ZnO. However, the Seebeck coefficient did not significantly change with morphology. The ZnO nanorods obtained the highest absolute value of the Seebeck coefficient in all temperature regions. This result indicates that ZnO nanorods will perform better in thermoelectric applications compared to other morphologies. In addition, electrical conductivity and thermal conductivity significantly varied with morphology and surface temperature. This, in turn, implies that the morphology and surface temperature are the main parameters in thermoelectric applications. All nanostructures’ conductivity increased as a function of temperature, which is attributed to stable morphology and surface temperature conditions. Therefore, there was a proportional relationship between conductivity and surface temperature. According to morphology, conductivity values considerably change (Figure 5). This may be due to the variation of mobility, carrier concentration, activation energy, crystallinity, and defects of different nanostructures. The ZnO nanorods showed the highest electrical conductivity values for all temperature ranges. This may be attributed to the higher carrier mobility due to the high aspect ratio, good crystallinity, lowest activation energy, and the lowest dominant defects of morphology. The power factor is another important parameter in thermoelectric applications. Generally, we have observed low thermal conductivity values for all morphologies, mainly due to the enhanced phonon scattering and the defects of the nanostructures. The variation in thermal conductivity may be attributed to the difference in grain size, grain boundary effect, and the defects of morphologies. Low thermal conductivity values are facilitated to improve the thermoelectric properties of ZnO nanostructures (Figure 6). We noted that the power factor values of nanostructures significantly increased (Figure 7) mainly due to the enhancement of electrical conductivity. By using the slope of the Arrhenius plot, we calculated the corresponding activation energy of all nanostructures (Figure 9). The change in activation energy imply the variation in energy levels corresponding to the conduction band and donor level in different nanostructures. The thermoelectric ZT values changes with morphology and surface temperature (Figure 8). Based on our work, the highest ZT value obtained in ZnO nanorods is 2.73 $\times 10^{-2}K^{-1}$, at 425 K. Hence, ZnO nanorods are an excellent candidate for thermoelectric applications and future work.

Based on our studies, we have observed excellent dielectric responses, seen in Figure 10, corresponding to different morphologies. The low-frequency dielectric constant is an artifact of impedance spectroscopy and is due to ions becoming trapped on the surfaces of the material. A more irregular and resistive surface should exhibit a higher dielectric constant in this region compared to high frequency, as seen by nanoparticles and nanoshuttles. Nanoshuttles have the lowest conductivity values, seen in Figure 4, followed by nanoparticles, and these two morphologies exhibit a second relaxation curve due to the charge buildup. In contrast, the smooth and conductive surfaces of nanoribbons and nanorods allow for charge carriers to flow easily and so the polarization in this region compared to high frequencies is relatively small. The high-frequency dielectric constant is within the range that is dominated by dipole polarization, and thus is more representative of the state inside the material itself, where recombination occurs. The pure crystalline structure of the ZnO nanorods compared to the other ZnO morphologies allows for atomic dipoles to align in the presence of an electric field more readily and neatly, thus leading to a higher dielectric constant within these frequencies. This dielectric constant is more relevant when choosing a material for photovoltaic applications. The low frequency dielectric constants of materials would indicate usefulness in capacitors and energy storage devices. According to our data, nanoshuttles provide the best properties for a dielectric material in a capacitor.

## 5. Conclusions

In summary, we have investigated the effect of morphology on thermoelectric, electrochemical, and dielectric properties of ZnO nanostructures, namely nanoribbons, nanorods, nanoparticles, and nanoshuttles. All ZnO morphologies show an enhancement pattern of the thermoelectric properties. According to the C-V results, the position of the conduction and valence band is considerably varied with morphology and the choice of optical filter wavelength used for excitation. If we control the solar spectrum with a red monochromic filter to a ZnO-based solar cell, it would give more output current due to the reduction of the bandgap. For all nanostructures, electrical conductivity values showed an increasing trend with increasing temperature from 325 K to 425 K range, which also confirmed the semiconducting behavior. In all ZnO morphologies, the highest conductivity value achieved by nanorods is 1097.60 Ω^−1^ m^−1^ at 425 K. The negative Seebeck coefficient of all morphologies verified that synthesized nanostructures have electrons as the leading transport carrier in nanostructured ZnO. Generally, the *PF* (Power factor) values of nanostructures increase with temperature, and the highest *PF* value obtained by nanorods is 3.19 $\times 10^{-4}\;K^{-1}$at 425 K. We observed changes in thermal conductivity for all nanostructures from 325 K to 425 K. The low thermal conductivity values of these ZnO morphologies are advantageous for thermoelectric applications. The ZnO nanorods have obtained the lowest activation energy 2.40 $\times 10^{-2}$ eV compared to all morphologies. The highest ZT was achieved in ZnO nanorods as 2.73 $\times 10^{-2}\;K^{-1}$ at 425 K, which implies that nanorods may be more suitable for good thermoelectric devices compared with other morphologies. The relatively high dielectric constant of ZnO nanorods at the MHz range compared to the other morphologies indicate that the recombination rate inside nanorods is lower, and is a much more favorable material for photovoltaic and thermoelectric applications in this regard. Overall, ZnO nanorods could achieve higher electrochemical, thermoelectric, and dielectric performance compared to all other morphologies. Therefore, nanorods are the most promising morphology for photovoltaic and thermoelectric applications, which was the main aim of this work. It is also evident from our analysis that by changing the morphologies of ZnO nanostructures, we can tune the electrical, thermal, and dielectric properties to the desired application.

## Figures and Tables

**Table 1:** Wavelengths of optical filters.

Optical Filters Color	Wavelength (nm)
Blue	493
Green	518
Yellow	572
Orange	595
Red	628

**Table 2:** C-V parameters of different ZnO nanostructures.

Nanostructure Name	Optical Filters Wavelength (nm)	$E_{HOMO}\;(\mathrm{eV})$	$E_{LUMO}\;(\mathrm{eV})$	Bandgap (eV)
Nanoribbons	493	−7.36	−4.03	3.34
	518	−7.33	−4.06	3.27
	572	−7.31	−4.06	3.25
	595	−7.29	−4.09	3.20
	628	−7.24	−4.14	3.10
	493	−6.98	−3.84	3.14
	518	−6.83	−3.83	3.00
Nanorods	572	−6.86	−3.86	3.00
	595	−6.86	−3.88	2.98
	628	−6.81	−3.90	2.91
	493	−7.31	−3.91	3.40
	518	−7.30	−3.93	3.37
Nanoparticles	572	−7.30	−3.96	3.34
	595	−7.30	−4.02	3.28
	628	−7.28	−4.12	3.16
Nanoshuttles	493	−7.17	−3.86	3.31
	518	−7.13	−3.88	3.25
	572	−7.09	−3.88	3.21
	595	−7.06	−3.90	3.16
	628	−6.93	−3.91	3.02

**Table 3:** Cole-Davidson parameters for nanoribbons and, nanorods.

Morphology	$\varepsilon_{\infty }$	$\varepsilon_{s}$	τ	β
Nanoribbons	1.21	2.01	1.21 $\times 10^{-4}$	2.11 $\times 10^{-1}$
Nanorods	2.02	5.21	4.66 $\times 10^{-4}$	6.53 $\times 10^{-1}$

**Table 4:** Cole—Davidson parameters for nanoparticles and nanoshuttles.

Morphology	$\varepsilon_{\infty }$	$\varepsilon_{i}$	$\tau_{i}\;(s)$	$\beta_{i}$	$\varepsilon_{s}$	$\tau_{s}\;(s)$	$\beta_{s}$
Nanoparticles	1.42	2.08	$9.92\times 10^{-6}$	$6.05\times 10^{-1}$	4.68	$2.64\times 10^{-3}s$	$2.25\times 10^{-1}$
Nanoshuttles	1.15	3.20	$1.28\times 10^{-5}$	$3.49\times 10^{-1}$	9.69	$7.57\times 10^{-4}s$	$8.53\times 10^{-1}$

## Data Availability

The datasets generated during this study are available from the corresponding author upon reasonable request.
